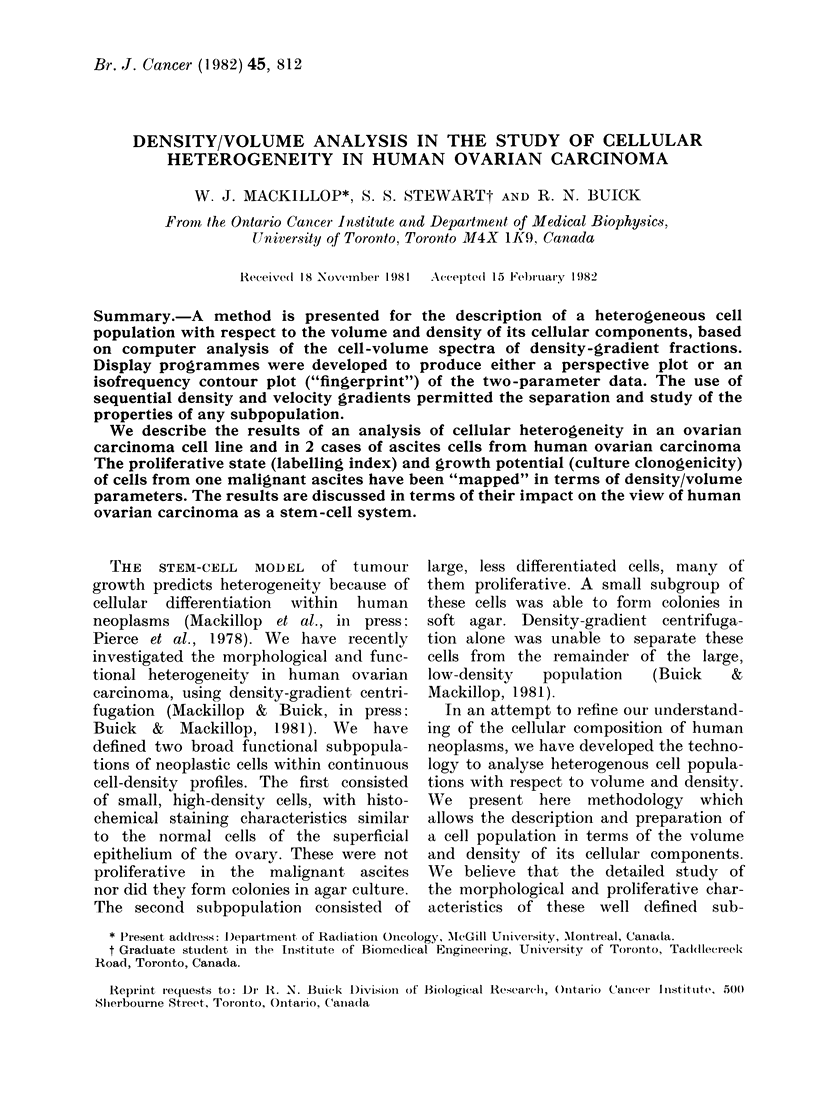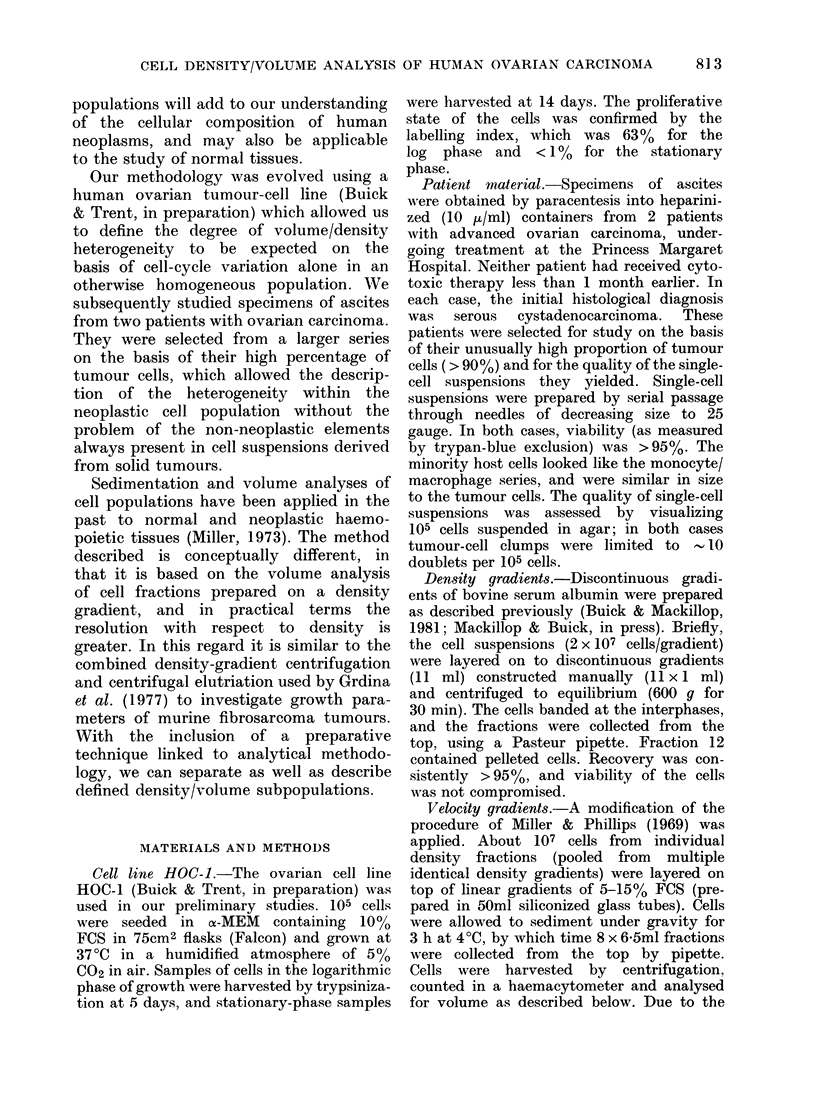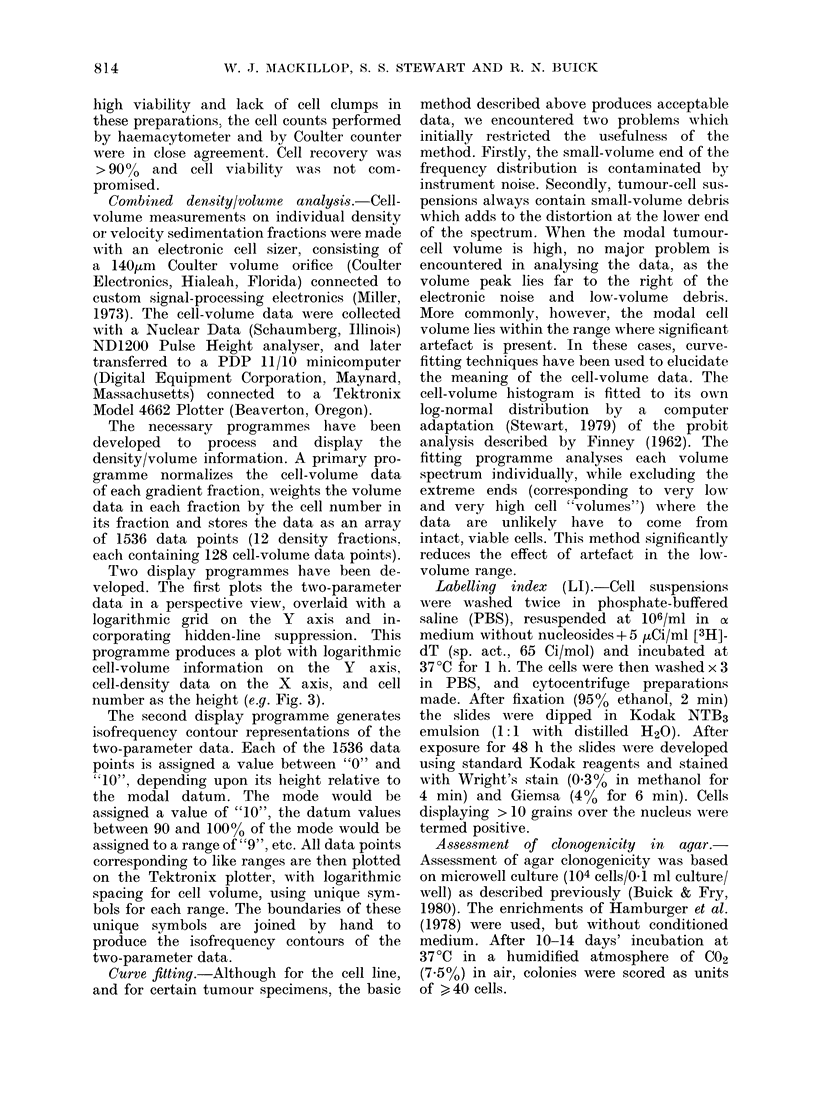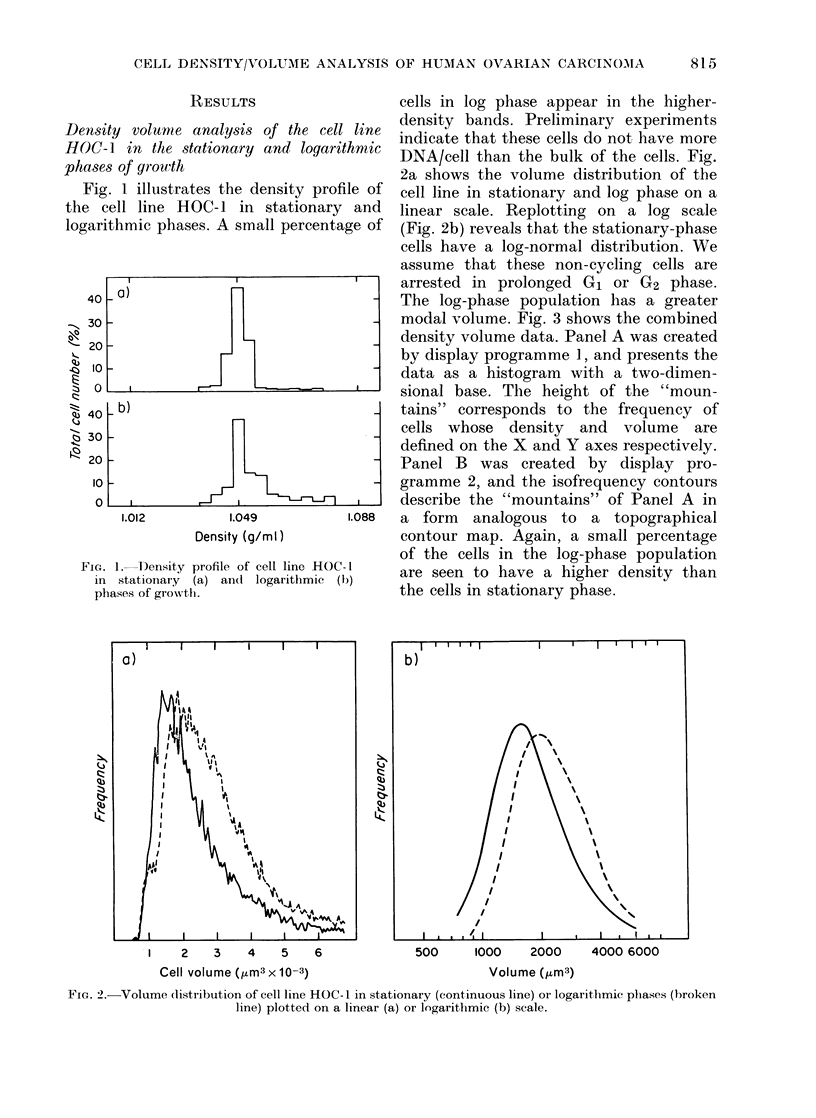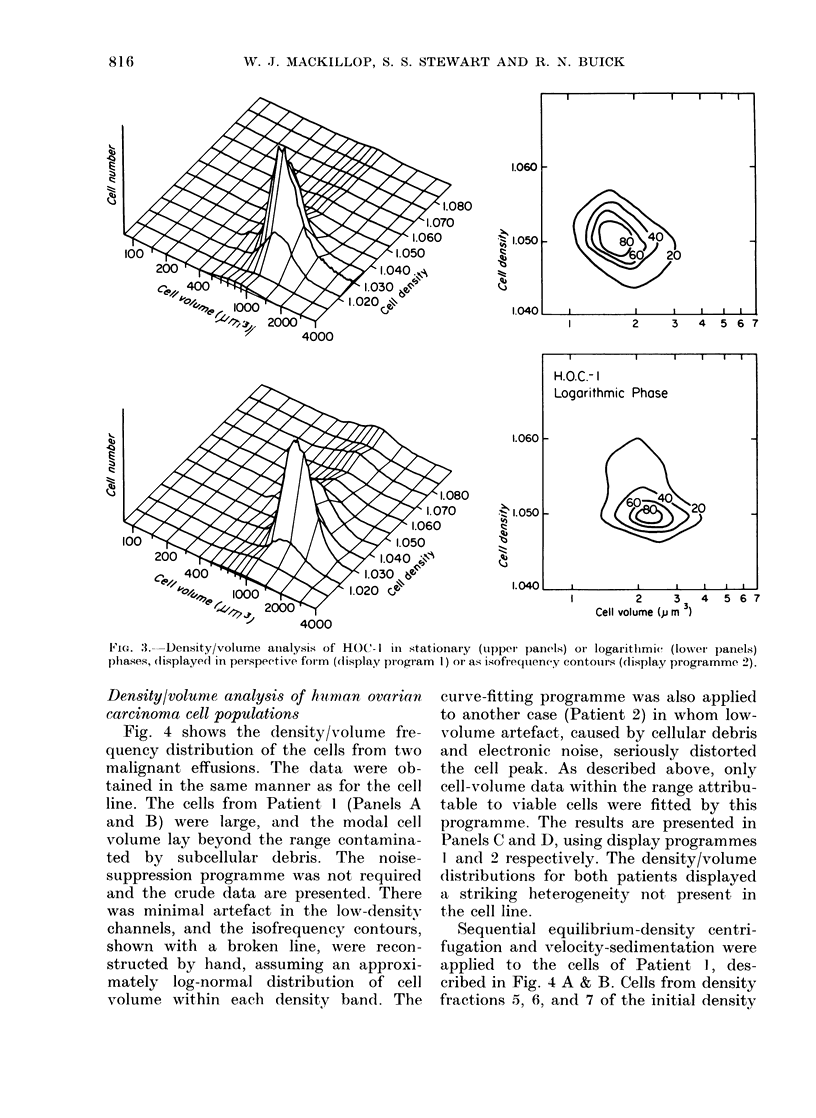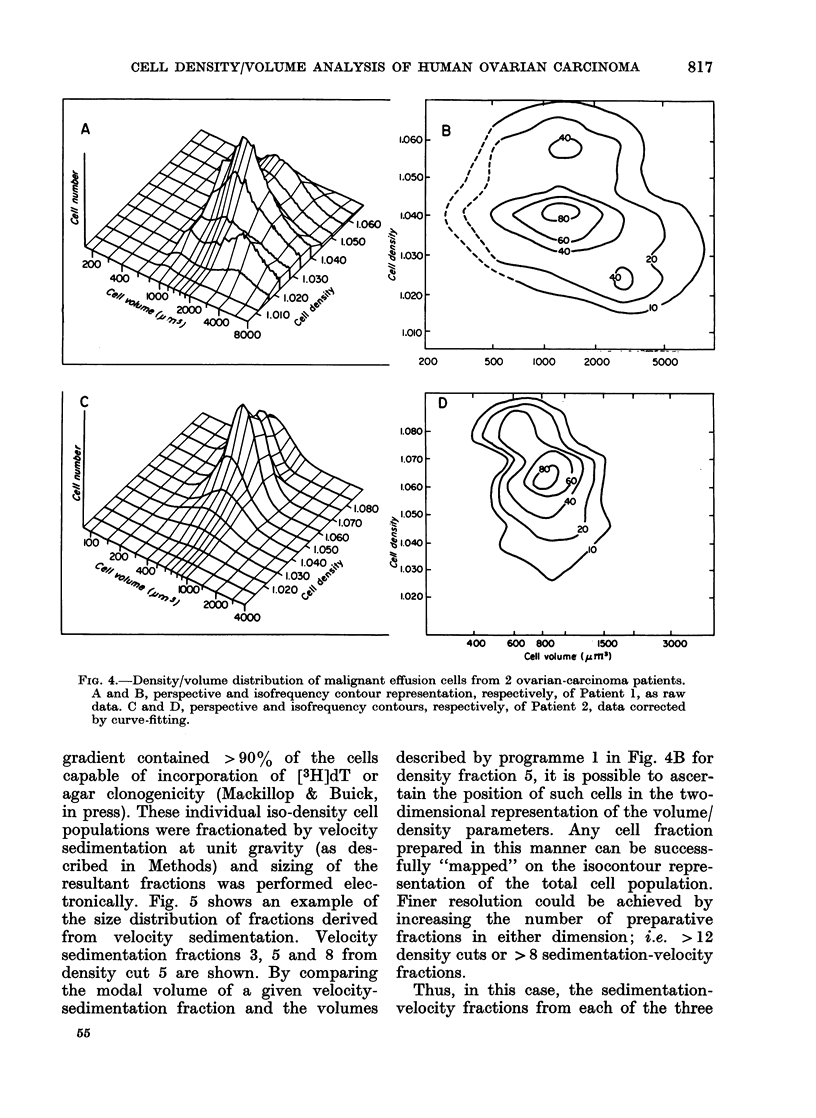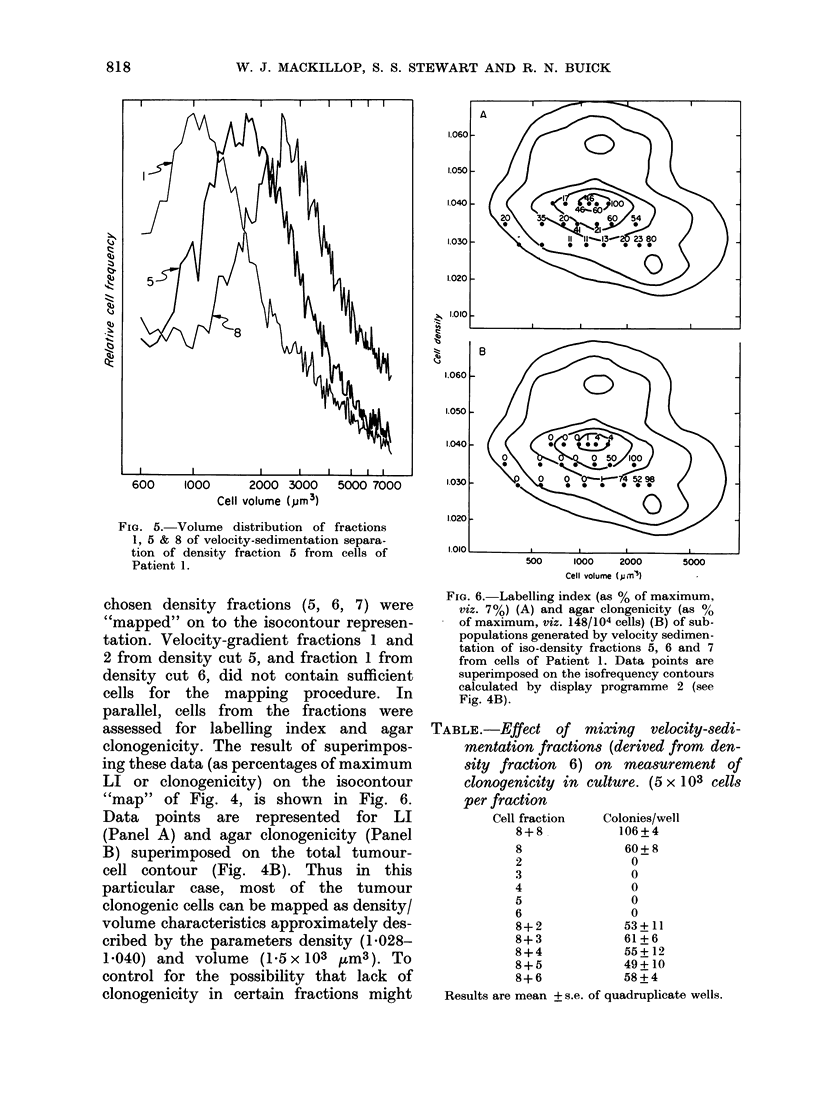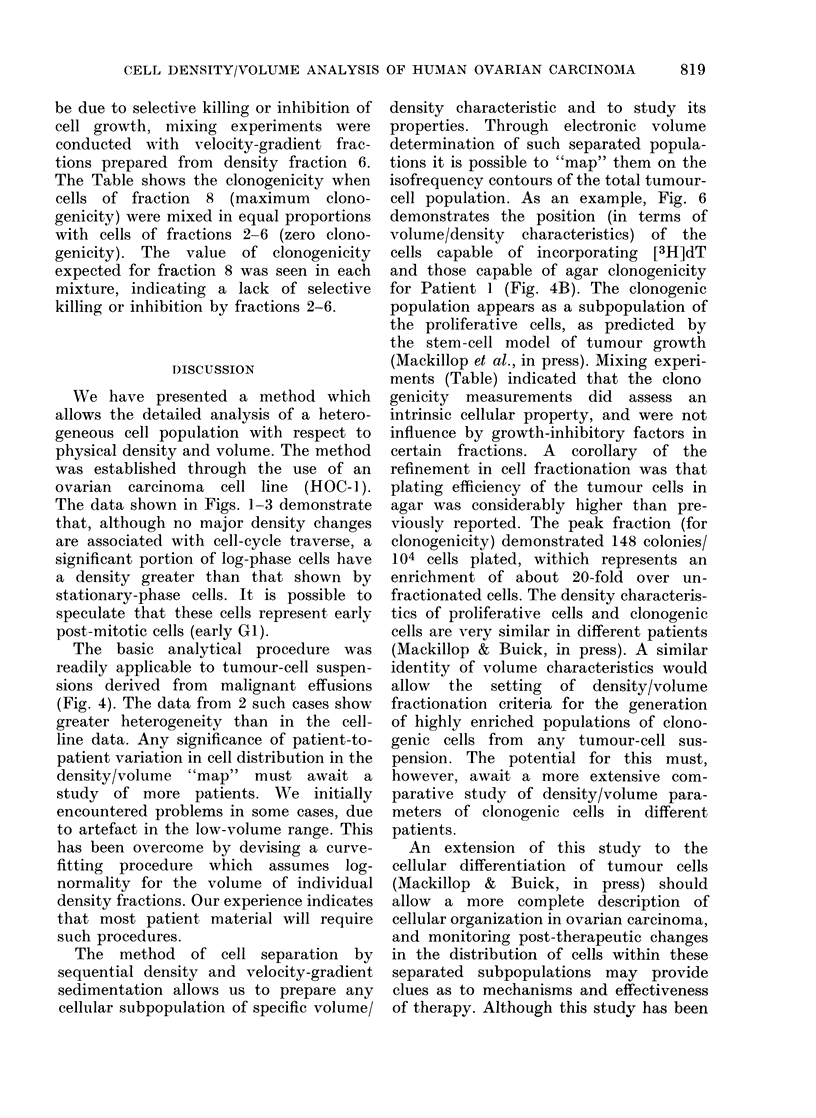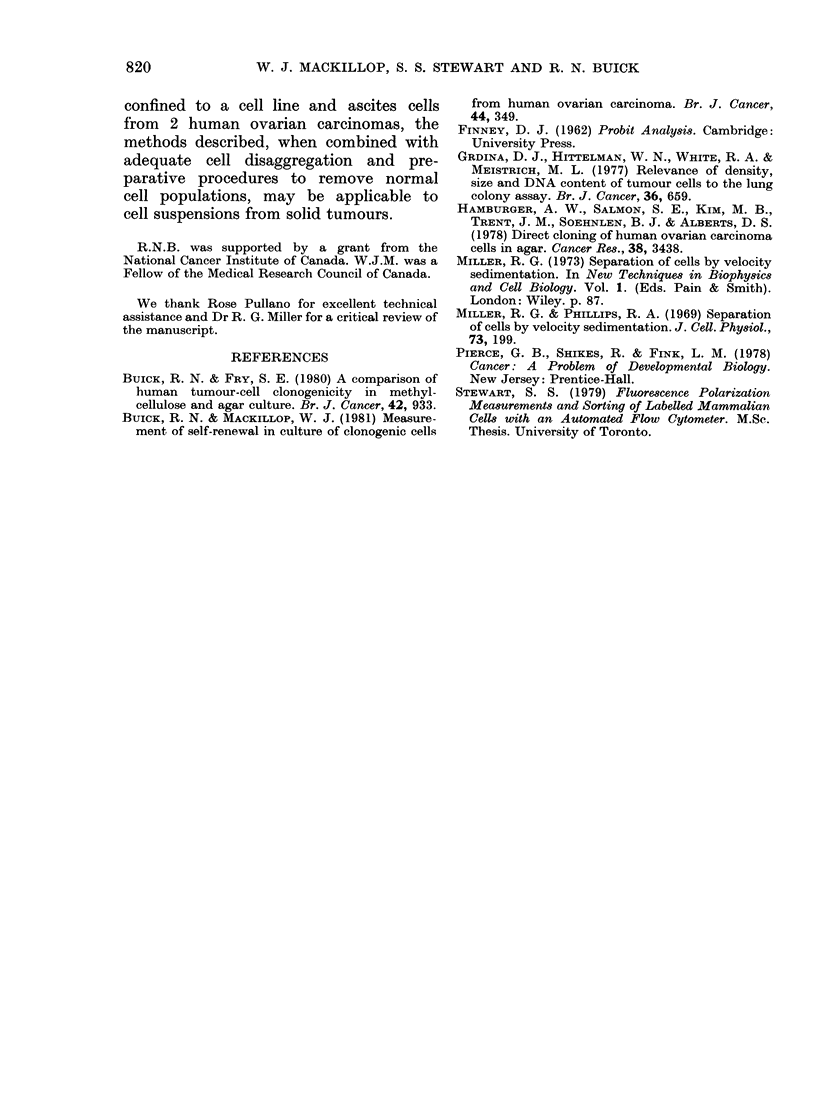# Density/volume analysis in the study of cellular heterogeneity in human ovarian carcinoma.

**DOI:** 10.1038/bjc.1982.132

**Published:** 1982-06

**Authors:** W. J. Mackillop, S. S. Stewart, R. N. Buick

## Abstract

A method is presented for the description of a heterogeneous cell population with respect to the volume and density of its cellular components, based on computer analysis of the cell-volume spectra of density-gradient fractions. Display programmes were developed to produce either a perspective plot or an isofrequency contour plot ("fingerprint") of the two-parameter data. The use of sequential density and velocity gradients permitted the separation and study of the properties of any subpopulation. We describe the results of an analysis of cellular heterogeneity in an ovarian carcinoma cell line and in 2 cases of ascites cells from human ovarian carcinoma. The proliferative state (labelling index) and growth potential (culture clonogenicity) of cells from one malignant ascites have been "mapped" in terms of density/volume parameters. The results are discussed in terms of their impact on the view of human ovarian carcinoma as a stem-cell system.


*Br. J. Cancer* (1982) **45**, 812

### DENSITY/VOLUME ANALYSIS IN THE STUDY OF CELLULAR HETEROGENEITY IN HUMAN OVARIAN CARCINOMA

W. J. MACKILLOP*, S. S. STEWART† AND R. N. BUICK

*From the Ontario Cancer Institute and Department of Medical Biophysics, University of Toronto, Toronto M4X 1K9, Canada*

Received 18 November 1981 Accepted 15 February 1982

**Summary.—A method is presented for the description of a heterogeneous cell population with respect to the volume and density of its cellular components, based on computer analysis of the cell-volume spectra of density-gradient fractions. Display programmes were developed to produce either a perspective plot or an isofrequency contour plot ("fingerprint") of the two-parameter data. The use of sequential density and velocity gradients permitted the separation and study of the properties of any subpopulation.**

**We describe the results of an analysis of cellular heterogeneity in an ovarian carcinoma cell line and in 2 cases of ascites cells from human ovarian carcinoma The proliferative state (labelling index) and growth potential (culture clonogenicity) of cells from one malignant ascites have been "mapped" in terms of density/volume parameters. The results are discussed in terms of their impact on the view of human ovarian carcinoma as a stem-cell system.**

THE STEM-CELL MODEL of tumour growth predicts heterogeneity because of cellular differentiation within human neoplasms (Mackillop *et al.*, in press; Pierce *et al.*, 1978). We have recently investigated the morphological and functional heterogeneity in human ovarian carcinoma, using density-gradient centrifugation (Mackillop & Buick, in press; Buick & Mackillop, 1981). We have defined two broad functional subpopulations of neoplastic cells within continuous cell-density profiles. The first consisted of small, high-density cells, with histochemical staining characteristics similar to the normal cells of the superficial epithelium of the ovary. These were not proliferative in the malignant ascites nor did they form colonies in agar culture. The second subpopulation consisted of large, less differentiated cells, many of them proliferative. A small subgroup of these cells was able to form colonies in soft agar. Density-gradient centrifugation alone was unable to separate these cells from the remainder of the large, low-density population (Buick & Mackillop, 1981).

In an attempt to refine our understanding of the cellular composition of human neoplasms, we have developed the technology to analyse heterogenous cell populations with respect to volume and density. We present here methodology which allows the description and preparation of a cell population in terms of the volume and density of its cellular components. We believe that the detailed study of the morphological and proliferative characteristics of these well defined sub-

* Present address: Department of Radiation Oncology, McGill University, Montreal, Canada.

† Graduate student in the Institute of Biomedical Engineering, University of Toronto, Taddlecreek Road, Toronto, Canada.

Reprint requests to: Dr R. N. Buick Division of Biological Research, Ontario Cancer Institute, 500 Sherbourne Street, Toronto, Ontario, Canada populations will add to our understanding of the cellular composition of human neoplasms, and may also be applicable to the study of normal tissues.

Our methodology was evolved using a human ovarian tumour-cell line (Buick & Trent, in preparation) which allowed us to define the degree of volume/density heterogeneity to be expected on the basis of cell-cycle variation alone in an otherwise homogeneous population. We subsequently studied specimens of ascites from two patients with ovarian carcinoma. They were selected from a larger series on the basis of their high percentage of tumour cells, which allowed the description of the heterogeneity within the neoplastic cell population without the problem of the non-neoplastic elements always present in cell suspensions derived from solid tumours.

Sedimentation and volume analyses of cell populations have been applied in the past to normal and neoplastic haemopoietic tissues (Miller, 1973). The method described is conceptually different, in that it is based on the volume analysis of cell fractions prepared on a density gradient, and in practical terms the resolution with respect to density is greater. In this regard it is similar to the combined density-gradient centrifugation and centrifugal elutriation used by Grdina *et al.* (1977) to investigate growth parameters of murine fibrosarcoma tumours. With the inclusion of a preparative technique linked to analytical methodology, we can separate as well as describe defined density/volume subpopulations.

### MATERIALS AND METHODS

*Cell line HOC-1.*—The ovarian cell line HOC-1 (Buick & Trent, in preparation) was used in our preliminary studies. $10^5$ cells were seeded in α-MEM containing 10% FCS in 75cm$^2$ flasks (Falcon) and grown at 37°C in a humidified atmosphere of 5% $CO_2$ in air. Samples of cells in the logarithmic phase of growth were harvested by trypsinization at 5 days, and stationary-phase samples were harvested at 14 days. The proliferative state of the cells was confirmed by the labelling index, which was 63% for the log phase and <1% for the stationary phase.

*Patient material.*—Specimens of ascites were obtained by paracentesis into heparinized (10 μ/ml) containers from 2 patients with advanced ovarian carcinoma, undergoing treatment at the Princess Margaret Hospital. Neither patient had received cytotoxic therapy less than 1 month earlier. In each case, the initial histological diagnosis was serous cystadenocarcinoma. These patients were selected for study on the basis of their unusually high proportion of tumour cells (>90%) and for the quality of the single-cell suspensions they yielded. Single-cell suspensions were prepared by serial passage through needles of decreasing size to 25 gauge. In both cases, viability (as measured by trypan-blue exclusion) was >95%. The minority host cells looked like the monocyte/macrophage series, and were similar in size to the tumour cells. The quality of single-cell suspensions was assessed by visualizing $10^5$ cells suspended in agar; in both cases tumour-cell clumps were limited to ~10 doublets per $10^5$ cells.

*Density gradients.*—Discontinuous gradients of bovine serum albumin were prepared as described previously (Buick & Mackillop, 1981; Mackillop & Buick, in press). Briefly, the cell suspensions ($2 \times 10^7$ cells/gradient) were layered on to discontinuous gradients (11 ml) constructed manually (11 × 1 ml) and centrifuged to equilibrium (600 *g* for 30 min). The cells banded at the interphases, and the fractions were collected from the top, using a Pasteur pipette. Fraction 12 contained pelleted cells. Recovery was consistently >95%, and viability of the cells was not compromised.

*Velocity gradients.*—A modification of the procedure of Miller & Phillips (1969) was applied. About $10^7$ cells from individual density fractions (pooled from multiple identical density gradients) were layered on top of linear gradients of 5–15% FCS (prepared in 50ml siliconized glass tubes). Cells were allowed to sediment under gravity for 3 h at 4°C, by which time 8 × 6·5ml fractions were collected from the top by pipette. Cells were harvested by centrifugation, counted in a haemacytometer and analysed for volume as described below. Due to the high viability and lack of cell clumps in these preparations, the cell counts performed by haemacytometer and by Coulter counter were in close agreement. Cell recovery was >90% and cell viability was not compromised.

*Combined density/volume analysis.*—Cell-volume measurements on individual density or velocity sedimentation fractions were made with an electronic cell sizer, consisting of a 140μm Coulter volume orifice (Coulter Electronics, Hialeah, Florida) connected to custom signal-processing electronics (Miller, 1973). The cell-volume data were collected with a Nuclear Data (Schaumberg, Illinois) ND1200 Pulse Height analyser, and later transferred to a PDP 11/10 minicomputer (Digital Equipment Corporation, Maynard, Massachusetts) connected to a Tektronix Model 4662 Plotter (Beaverton, Oregon).

The necessary programmes have been developed to process and display the density/volume information. A primary programme normalizes the cell-volume data of each gradient fraction, weights the volume data in each fraction by the cell number in its fraction and stores the data as an array of 1536 data points (12 density fractions, each containing 128 cell-volume data points).

Two display programmes have been developed. The first plots the two-parameter data in a perspective view, overlaid with a logarithmic grid on the Y axis and incorporating hidden-line suppression. This programme produces a plot with logarithmic cell-volume information on the Y axis, cell-density data on the X axis, and cell number as the height (*e.g.* Fig. 3).

The second display programme generates isofrequency contour representations of the two-parameter data. Each of the 1536 data points is assigned a value between "0" and "10", depending upon its height relative to the modal datum. The mode would be assigned a value of "10", the datum values between 90 and 100% of the mode would be assigned to a range of "9", etc. All data points corresponding to like ranges are then plotted on the Tektronix plotter, with logarithmic spacing for cell volume, using unique symbols for each range. The boundaries of these unique symbols are joined by hand to produce the isofrequency contours of the two-parameter data.

*Curve fitting.*—Although for the cell line, and for certain tumour specimens, the basic method described above produces acceptable data, we encountered two problems which initially restricted the usefulness of the method. Firstly, the small-volume end of the frequency distribution is contaminated by instrument noise. Secondly, tumour-cell suspensions always contain small-volume debris which adds to the distortion at the lower end of the spectrum. When the modal tumour-cell volume is high, no major problem is encountered in analysing the data, as the volume peak lies far to the right of the electronic noise and low-volume debris. More commonly, however, the modal cell volume lies within the range where significant artefact is present. In these cases, curve-fitting techniques have been used to elucidate the meaning of the cell-volume data. The cell-volume histogram is fitted to its own log-normal distribution by a computer adaptation (Stewart, 1979) of the probit analysis described by Finney (1962). The fitting programme analyses each volume spectrum individually, while excluding the extreme ends (corresponding to very low and very high cell "volumes") where the data are unlikely have to come from intact, viable cells. This method significantly reduces the effect of artefact in the low-volume range.

*Labelling index* (LI).—Cell suspensions were washed twice in phosphate-buffered saline (PBS), resuspended at $10^6$/ml in α medium without nucleosides + 5 μCi/ml [$^3$H]-dT (sp. act., 65 Ci/mol) and incubated at 37°C for 1 h. The cells were then washed ×3 in PBS, and cytocentrifuge preparations made. After fixation (95% ethanol, 2 min) the slides were dipped in Kodak $NTB_3$ emulsion (1:1 with distilled $H_2O$). After exposure for 48 h the slides were developed using standard Kodak reagents and stained with Wright's stain (0·3% in methanol for 4 min) and Giemsa (4% for 6 min). Cells displaying >10 grains over the nucleus were termed positive.

*Assessment of clonogenicity in agar.*—Assessment of agar clonogenicity was based on microwell culture ($10^4$ cells/0·1 ml culture/well) as described previously (Buick & Fry, 1980). The enrichments of Hamburger *et al.* (1978) were used, but without conditioned medium. After 10–14 days' incubation at 37°C in a humidified atmosphere of $CO_2$ (7·5%) in air, colonies were scored as units of ⩾40 cells.

### Results

*Density volume analysis of the cell line HOC-1 in the stationary and logarithmic phases of growth*

Fig. 1 illustrates the density profile of the cell line HOC-1 in stationary and logarithmic phases. A small percentage of cells in log phase appear in the higher-density bands. Preliminary experiments indicate that these cells do not have more DNA/cell than the bulk of the cells. Fig. 2a shows the volume distribution of the cell line in stationary and log phase on a linear scale. Replotting on a log scale (Fig. 2b) reveals that the stationary-phase cells have a log-normal distribution. We assume that these non-cycling cells are arrested in prolonged $G_1$ or $G_2$ phase. The log-phase population has a greater modal volume. Fig. 3 shows the combined density volume data. Panel A was created by display programme 1, and presents the data as a histogram with a two-dimensional base. The height of the "mountains" corresponds to the frequency of cells whose density and volume are defined on the X and Y axes respectively. Panel B was created by display programme 2, and the isofrequency contours describe the "mountains" of Panel A in a form analogous to a topographical contour map. Again, a small percentage of the cells in the log-phase population are seen to have a higher density than the cells in stationary phase.

FIG. 1.—Density profile of cell line HOC-1 in stationary (a) and logarithmic (b) phases of growth.

FIG. 2.—Volume distribution of cell line HOC-1 in stationary (continuous line) or logarithmic phases (broken line) plotted on a linear (a) or logarithmic (b) scale.

FIG. 3.—Density/volume analysis of HOC-1 in stationary (upper panels) or logarithmic (lower panels) phases, displayed in perspective form (display program 1) or as isofrequency contours (display programme 2).

*Density/volume analysis of human ovarian carcinoma cell populations*

Fig. 4 shows the density/volume frequency distribution of the cells from two malignant effusions. The data were obtained in the same manner as for the cell line. The cells from Patient 1 (Panels A and B) were large, and the modal cell volume lay beyond the range contaminated by subcellular debris. The noise-suppression programme was not required and the crude data are presented. There was minimal artefact in the low-density channels, and the isofrequency contours, shown with a broken line, were reconstructed by hand, assuming an approximately log-normal distribution of cell volume within each density band. The curve-fitting programme was also applied to another case (Patient 2) in whom low-volume artefact, caused by cellular debris and electronic noise, seriously distorted the cell peak. As described above, only cell-volume data within the range attributable to viable cells were fitted by this programme. The results are presented in Panels C and D, using display programmes 1 and 2 respectively. The density/volume distributions for both patients displayed a striking heterogeneity not present in the cell line.

Sequential equilibrium-density centrifugation and velocity-sedimentation were applied to the cells of Patient 1, described in Fig. 4 A & B. Cells from density fractions 5, 6, and 7 of the initial density

FIG. 4.—Density/volume distribution of malignant effusion cells from 2 ovarian-carcinoma patients. A and B, perspective and isofrequency contour representation, respectively, of Patient 1, as raw data. C and D, perspective and isofrequency contours, respectively, of Patient 2, data corrected by curve-fitting.

gradient contained >90% of the cells capable of incorporation of [$^3$H]dT or agar clonogenicity (Mackillop & Buick, in press). These individual iso-density cell populations were fractionated by velocity sedimentation at unit gravity (as described in Methods) and sizing of the resultant fractions was performed electronically. Fig. 5 shows an example of the size distribution of fractions derived from velocity sedimentation. Velocity sedimentation fractions 3, 5 and 8 from density cut 5 are shown. By comparing the modal volume of a given velocity-sedimentation fraction and the volumes described by programme 1 in Fig. 4B for density fraction 5, it is possible to ascertain the position of such cells in the two-dimensional representation of the volume/density parameters. Any cell fraction prepared in this manner can be successfully "mapped" on the isocontour representation of the total cell population. Finer resolution could be achieved by increasing the number of preparative fractions in either dimension; *i.e.* >12 density cuts or >8 sedimentation-velocity fractions.

Thus, in this case, the sedimentation-velocity fractions from each of the three

FIG. 5.—Volume distribution of fractions 1, 5 & 8 of velocity-sedimentation separation of density fraction 5 from cells of Patient 1.

FIG. 6.—Labelling index (as % of maximum, *viz.* 7%) (A) and agar clongenicity (as % of maximum, *viz.* $148/10^4$ cells) (B) of subpopulations generated by velocity sedimentation of iso-density fractions 5, 6 and 7 from cells of Patient 1. Data points are superimposed on the isofrequency contours calculated by display programme 2 (see Fig. 4B).

chosen density fractions (5, 6, 7) were "mapped" on to the isocontour representation. Velocity-gradient fractions 1 and 2 from density cut 5, and fraction 1 from density cut 6, did not contain sufficient cells for the mapping procedure. In parallel, cells from the fractions were assessed for labelling index and agar clonogenicity. The result of superimposing these data (as percentages of maximum LI or clonogenicity) on the isocontour "map" of Fig. 4, is shown in Fig. 6. Data points are represented for LI (Panel A) and agar clonogenicity (Panel B) superimposed on the total tumour-cell contour (Fig. 4B). Thus in this particular case, most of the tumour clonogenic cells can be mapped as density/volume characteristics approximately described by the parameters density (1·028–1·040) and volume ($1{\cdot}5 \times 10^3$ $\mu m^3$). To control for the possibility that lack of clonogenicity in certain fractions might

TABLE.—*Effect of mixing velocity-sedimentation fractions (derived from density fraction 6) on measurement of clonogenicity in culture.* ($5 \times 10^3$ *cells per fraction*

| Cell fraction | Colonies/well |
|---|---|
| 8+8 | 106±4 |
| 8 | 60±8 |
| 2 | 0 |
| 3 | 0 |
| 4 | 0 |
| 5 | 0 |
| 6 | 0 |
| 8+2 | 53±11 |
| 8+3 | 61±6 |
| 8+4 | 55±12 |
| 8+5 | 49±10 |
| 8+6 | 58±4 |

Results are mean ±s.e. of quadruplicate wells.

be due to selective killing or inhibition of cell growth, mixing experiments were conducted with velocity-gradient fractions prepared from density fraction 6. The Table shows the clonogenicity when cells of fraction 8 (maximum clonogenicity) were mixed in equal proportions with cells of fractions 2–6 (zero clonogenicity). The value of clonogenicity expected for fraction 8 was seen in each mixture, indicating a lack of selective killing or inhibition by fractions 2–6.

### DISCUSSION

We have presented a method which allows the detailed analysis of a heterogeneous cell population with respect to physical density and volume. The method was established through the use of an ovarian carcinoma cell line (HOC-1). The data shown in Figs. 1–3 demonstrate that, although no major density changes are associated with cell-cycle traverse, a significant portion of log-phase cells have a density greater than that shown by stationary-phase cells. It is possible to speculate that these cells represent early post-mitotic cells (early G1).

The basic analytical procedure was readily applicable to tumour-cell suspensions derived from malignant effusions (Fig. 4). The data from 2 such cases show greater heterogeneity than in the cell-line data. Any significance of patient-to-patient variation in cell distribution in the density/volume "map" must await a study of more patients. We initially encountered problems in some cases, due to artefact in the low-volume range. This has been overcome by devising a curve-fitting procedure which assumes log-normality for the volume of individual density fractions. Our experience indicates that most patient material will require such procedures.

The method of cell separation by sequential density and velocity-gradient sedimentation allows us to prepare any cellular subpopulation of specific volume/density characteristic and to study its properties. Through electronic volume determination of such separated populations it is possible to "map" them on the isofrequency contours of the total tumour-cell population. As an example, Fig. 6 demonstrates the position (in terms of volume/density characteristics) of the cells capable of incorporating [$^3$H]dT and those capable of agar clonogenicity for Patient 1 (Fig. 4B). The clonogenic population appears as a subpopulation of the proliferative cells, as predicted by the stem-cell model of tumour growth (Mackillop *et al.*, in press). Mixing experiments (Table) indicated that the clono genicity measurements did assess an intrinsic cellular property, and were not influence by growth-inhibitory factors in certain fractions. A corollary of the refinement in cell fractionation was that plating efficiency of the tumour cells in agar was considerably higher than previously reported. The peak fraction (for clonogenicity) demonstrated 148 colonies/$10^4$ cells plated, withich represents an enrichment of about 20-fold over unfractionated cells. The density characteristics of proliferative cells and clonogenic cells are very similar in different patients (Mackillop & Buick, in press). A similar identity of volume characteristics would allow the setting of density/volume fractionation criteria for the generation of highly enriched populations of clonogenic cells from any tumour-cell suspension. The potential for this must, however, await a more extensive comparative study of density/volume parameters of clonogenic cells in different patients.

An extension of this study to the cellular differentiation of tumour cells (Mackillop & Buick, in press) should allow a more complete description of cellular organization in ovarian carcinoma, and monitoring post-therapeutic changes in the distribution of cells within these separated subpopulations may provide clues as to mechanisms and effectiveness of therapy. Although this study has been confined to a cell line and ascites cells from 2 human ovarian carcinomas, the methods described, when combined with adequate cell disaggregation and preparative procedures to remove normal cell populations, may be applicable to cell suspensions from solid tumours.

R.N.B. was supported by a grant from the National Cancer Institute of Canada. W.J.M. was a Fellow of the Medical Research Council of Canada.

We thank Rose Pullano for excellent technical assistance and Dr R. G. Miller for a critical review of the manuscript.

### REFERENCES

BUICK, R. N. & FRY, S. E. (1980) A comparison of human tumour-cell clonogenicity in methylcellulose and agar culture. *Br. J. Cancer*, **42**, 933.

BUICK, R. N. & MACKILLOP, W. J. (1981) Measurement of self-renewal in culture of clonogenic cells from human ovarian carcinoma. *Br. J. Cancer*, **44**, 349.

FINNEY, D. J. (1962) *Probit Analysis.* Cambridge: University Press.

GRDINA, D. J., HITTELMAN, W. N., WHITE, R. A. & MEISTRICH, M. L. (1977) Relevance of density, size and DNA content of tumour cells to the lung colony assay. *Br. J. Cancer*, **36**, 659.

HAMBURGER, A. W., SALMON, S. E., KIM, M. B., TRENT, J. M., SOEHNLEN, B. J. & ALBERTS, D. S. (1978) Direct cloning of human ovarian carcinoma cells in agar. *Cancer Res.*, **38**, 3438.

MILLER, R. G. (1973) Separation of cells by velocity sedimentation. In *New Techniques in Biophysics and Cell Biology.* Vol. **1**. (Eds. Pain & Smith). London: Wiley. p. 87.

MILLER, R. G. & PHILLIPS, R. A. (1969) Separation of cells by velocity sedimentation. *J. Cell. Physiol.*, **73**, 199.

PIERCE, G. B., SHIKES, R. & FINK, L. M. (1978) *Cancer: A Problem of Developmental Biology.* New Jersey: Prentice-Hall.

STEWART, S. S. (1979) *Fluorescence Polarization Measurements and Sorting of Labelled Mammalian Cells with an Automated Flow Cytometer.* M.Sc. Thesis. University of Toronto.